# Extrapulmonary Tuberculosis in the Bone Marrow: A Case of Pancytopenia and Chronic Corticosteroid Use

**DOI:** 10.1155/carm/7747893

**Published:** 2025-11-18

**Authors:** Seyed Amirhossein Salehi, Minoo Heidari Almasi, Hamideh Moradi Shahrebabak, Farahnaz Bidari Zerehpoosh, Masih falahatian, Afsaneh Safarian

**Affiliations:** ^1^Student Research Committee, School of Medicine, Shahid Beheshti University of Medical Sciences, Tehran, Iran; ^2^Imam Hossein Hospital Clinical Research Development Unit, Shahid Beheshti University of Medical Sciences, Tehran, Iran; ^3^Loghman Hakim Hospital, SBMU, Tehran, Iran; ^4^Department of Radiology, Loghman Hakim Hospital, Shahid Beheshti University of Medical Sciences, Tehran, Iran; ^5^Department of Internal Medicine, Loghman Hakim Hospital, Shahid Beheshti University of Medical Science, Tehran, Iran

**Keywords:** bone marrow tuberculosis, caseating granulomas, chronic corticosteroid use, extrapulmonary tuberculosis, *Mycobacterium tuberculosis*, pancytopenia

## Abstract

**Background:**

Bone marrow tuberculosis (TB) is a rare but severe form of extrapulmonary TB, often presenting with nonspecific symptoms such as fatigue, weakness, and cytopenia, making diagnosis challenging.

**Case Presentation:**

We report the case of a 71-year-old male with a history of pituitary surgery and chronic corticosteroid use, who presented with pancytopenia, hyponatremia, and a chronic productive cough. Initial investigations, including chest CT, abdominal and pelvic ultrasound, liver elastography, dynamic liver MRI, blood cultures (from peripheral blood), and sputum cultures (from expectorated sputum), were inconclusive. A bone marrow biopsy revealed caseating granulomas, and acid-fast staining confirmed *Mycobacterium tuberculosis*.

**Management and Outcome:**

The patient was initiated on a 12-month antitubercular therapy regimen, extended due to the disseminated nature of bone marrow TB and the patient's immunocompromised state from chronic corticosteroid use, leading to clinical improvement and resolution of cytopenia.

**Discussion:**

This case highlights the diagnostic complexities of bone marrow TB, particularly in immunocompromised individuals, and underscores the importance of considering TB in patients with unexplained hematological abnormalities. Limited literature on this condition emphasizes the need for further research to enhance diagnostic accuracy and optimize treatment strategies.

**Conclusion:**

Early recognition and appropriate management are crucial for improving outcomes in this rare but serious manifestation of TB.

**Summary**

• Bone marrow tuberculosis (TB) is a rare but severe extrapulmonary TB form, often presenting with nonspecific symptoms such as pancytopenia and fatigue.
• Diagnosis is challenging and requires high suspicion, especially in immunocompromised patients.
• Early recognition and appropriate antitubercular therapy are crucial for improving outcomes.

## 1. Introduction

TB is a chronic infectious disease primarily caused by the bacterium *Mycobacterium tuberculosi s* (MTB). Although it predominantly affects the lungs, leading to pulmonary TB, it can also involve extrapulmonary sites, resulting in a wide range of clinical manifestations [1]. TB is a significant global health concern, with millions of cases reported annually, particularly in resource-limited settings. The disease is transmitted through airborne particles when an infected individual coughs or sneezes, making it a highly contagious condition [2]. Factors such as immunocompromised states, malnutrition, and socioeconomic determinants play a crucial role in the transmission and progression of TB, challenging public health efforts aimed at containment and eradication [3].

Bone marrow TB, an uncommon but serious form of extrapulmonary TB, occurs when the mycobacteria infiltrate the bone marrow, affecting its ability to produce blood cells effectively. This condition may manifest with a diverse set of symptoms, including anemia, leukopenia, and thrombocytopenia, due to the compromised hematopoietic function of the marrow [4]. Diagnosing bone marrow TB can be particularly challenging, as it often presents with nonspecific clinical findings and may mimic other hematological disorders. Advanced imaging techniques can identify suggestive findings, and bone marrow biopsy is essential in confirmatory diagnosis [5]. The complexity of its presentation necessitates a high index of suspicion and awareness among healthcare providers, especially in populations where TB is endemic. Treatment typically involves a rigorous regimen of anti-TB medications, which can be complicated by the presence of coexisting conditions such as HIV infection, further emphasizing the need for coordinated care in affected patients [6].

Here, we present to you, the case of a 71-year-old male, who was diagnosed with bone marrow TB.

## 2. Case Presentation

A 71-year-old man presented to the emergency department of Loqman Hakim Hospital, located in Tehran, Iran, complaining of significant weakness and fatigue that had been affecting his daily life. He had a notable medical history, specifically undergoing pituitary surgery due to a benign pituitary tumor. Following that surgery 5 years prior, he had been prescribed a daily dose of 5 mg prednisone to manage his condition. Upon his arrival at the hospital, the patient was afebrile, but he reported experiencing a chronic productive cough that had persisted for more than 3 months.

During the physical examination, his abdomen was found to be soft, and no signs of tachypnea, cyanosis, use of accessory muscles, or audible wheezing were observed. The patient was alert and oriented to person, place, and time, appearing well-nourished and euvolemic with no signs of dehydration. He was in no acute distress. Vital signs were stable. Abdominal examination showed a soft, nontender abdomen with normal bowel sounds, no guarding or rebound tenderness, and no hepatosplenomegaly on palpation (liver edge not palpable below the costal margin). The pulmonary examination was normal, with symmetric chest expansion, clear breath sounds bilaterally, and no wheezes, rales, rhonchi, or decreased air entry. No clubbing or cyanosis was noted. Extremities showed no edema, and neurological exam was intact with no focal deficits.

Initial laboratory results revealed pancytopenia—characterized by a white blood cell count of 3000 cells/μL (reference range: 4500–11,000 cells/μL), hemoglobin of 7.3 g/dL (reference range: 13.5–17.5 g/dL), and platelet count of 85,000 cells/μL (reference range: 150,000–450,000 cells/μL), indicative of bone marrow suppression likely due to MTB infiltration. Additionally, hyponatremia was noted (sodium level = 130 mmol/L—mild hyponatremia), with a serum sodium level below the normal range (typically < 135 mmol/L). Due to the severe anemia (hemoglobin 7.3 mg/dL), the patient received two units of packed red blood cells during hospitalization to stabilize his condition. It is worth noting that no prior medical history or laboratory records were available for the patient, which complicated the diagnostic process. The patient was interviewed regarding possible sources of TB exposure, including contacts with active TB cases (e.g., household members, coworkers, or community exposures), but no such contacts were reported. Interestingly, the patient mentioned that he regularly consumed unpasteurized dairy products, specifically unpasteurized milk and yogurt, which are common in Iranian cuisine and may represent a risk factor for zoonotic TB transmission via *Mycobacterium bovis*.

A comprehensive sepsis workup was initiated to investigate the underlying cause of his symptoms including blood cultures (obtained from peripheral blood), sputum cultures (from expectorated sputum), complete blood count (CBC), C-reactive protein (CRP), and serum electrolyte panel, and further imaging studies were ordered. Also, HIV testing was done, which came back negative. The results from a chest computed tomography (CT) scan, as well as abdominal and pelvic ultrasound, provided additional insights into his condition. Fiberoptic bronchoscopy and bronchoalveolar lavage were not performed, given the mild and nonspecific findings, stable respiratory status, and prioritization of bone marrow evaluation for pancytopenia. In an attempt to address his cytopenia, he was empirically treated with vitamin B12; however, this intervention did not lead to any improvements in his blood cell counts. A peripheral blood smear was conducted, and the morphology was reported as normal, further complicating the clinical picture.

### 2.1. Differential Diagnoses, Investigations, and Treatment

#### 2.1.1. CT Scan Report

• Liver: Several small hypodense lesions, too small to characterize, were observed in the right lobe of the liver. The gallbladder appears contracted and contains several stones.
• Spine: Degenerative changes are observed in the lumbar spine.
• Lungs and Pleura: Mild right-sided pleural effusion and minimal left-sided pleural effusion are seen. Several nodules are noted in the right lung, the largest measuring 6 mm in the right middle lobe (RML).

The lab findings are mentioned in Table 1.

As time progressed during his hospitalization, the patient's cytopenia appeared to worsen, prompting further diagnostic evaluation. Elastography of the liver was performed, followed by a dynamic MRI of the liver, to assess for any underlying hepatic conditions. Microbiological cultures taken during this period returned negative results, which ruled out certain infectious etiologies.

#### 2.1.2. Elastography Report

• Mean kPa = 11.5, which corresponds to a F3 METAVIR score.
• Liver Imaging Findings: Three hypoechoic lesions with central echogenic areas and a target-like appearance were observed in the right lobe of the liver. Further evaluation with dynamic contrast-enhanced MRI of the liver is recommended. The liver appears normal in size with slightly heterogeneous parenchymal echotexture. The hepatic veins and intrahepatic bile ducts are normal in diameter. Common bile duct (CBD) is 5.5 mm, and portal vein (PV) is 11 mm.
• Spleen: The spleen appears enlarged, measuring 145 mm in span (larger than normal). Multiple hypoechoic nodules are noted within the spleen. Further investigation for lymphoproliferative or infectious diseases is recommended.
• Gallbladder: The gallbladder is normal in size and wall thickness, containing several stones, the largest measuring 10 mm.

#### 2.1.3. MRI Report

**Figure 1:**
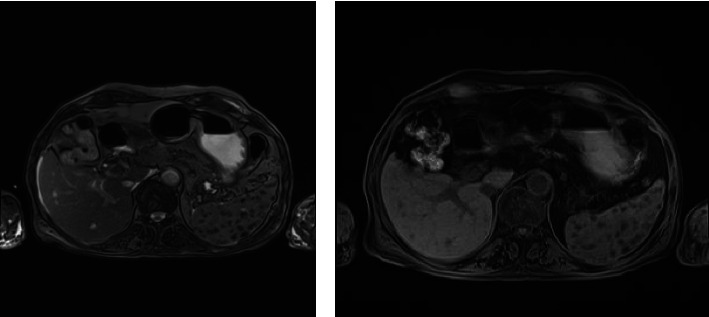
Multiple small T1 low/T2 high signal lesions are seen in the liver up to 6 mm with mild peripheral enhancement and target shape, numerous small T1 and T2 low signal foci without enhancement are seen in the spleen.

• Liver: Multiple small lesions are observed, measuring up to 6 mm, demonstrating T1 hypointense and T2 hyperintense signals. These lesions exhibit mild peripheral enhancement and a target-like appearance on ultrasonography, suggestive of microabscesses, likely secondary to fungal infection. Additionally, multiple small gallstones are noted (Figure 1).
• Spleen: Numerous small T1 and T2 hypointense foci are seen without significant enhancement. These findings raise the possibility of diffuse fungal infection, although lymphoma remains a less likely differential diagnosis. Splenomegaly is evident, with the spleen measuring 138 mm in size.
• Pleura: Bilateral mild pleural effusions are present, more prominent on the right side.

Small hypodense lesions in the liver were noted on CT but were further characterized by subsequent MRI. Liver elastography indicated an F3 METAVIR score (mean kPa = 11.5), reflecting moderate fibrosis, and showed three hypoechoic lesions with central echogenic areas and a target-like appearance in the right lobe. The dynamic liver MRI revealed multiple small lesions up to 6 mm with T1 hypointense/T2 hyperintense signals and mild peripheral enhancement, suggestive of microabscesses (likely fungal), though differentials included infectious (bacterial, mycobacterial, and parasitic), malignant (metastases, HCC, lymphoma), benign (hemangiomas, cysts), or inflammatory causes.

### 2.2. Outcome and Follow-Up

**Figure 2:**
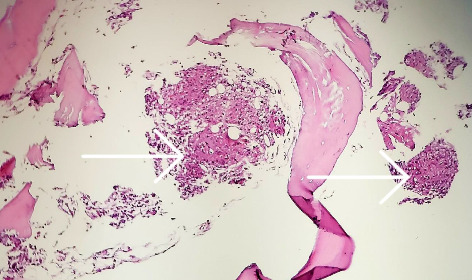
Trabecular bone with multiple granulomas in marrow spaces, two of them showed with arrow, H&E staining, × 100.

**Figure 3:**
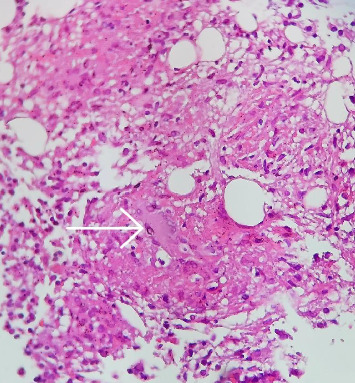
A granuloma with aggregates of epithelioid histiocytes and a multinucleated giant cell (arrow), H&E stain, × 400.

In view of the persistent cytopenia and the need for a definitive diagnosis, the patient underwent a bone marrow biopsy and aspiration. The morphologic examination and flow cytometry results of the bone marrow were reported as normal, which was promising. However, a granulomatous response with caseation was observed in the biopsy results, raising new concerns (Figures 2 and 3).

**Figure 4:**
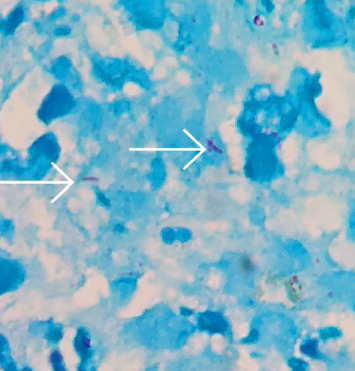
Acid-fast bacilli (*Mycobacterium tuberculosis*) revealed by arrows, Ziehl–Neelsen stain, × 1000.

The presence of *Mycobacterium* was confirmed through acid-fast staining, leading to the diagnosis of TB as the underlying cause of his symptoms (Figure 4). The sample culture for TB, further confirmed the diagnosis, but we did not delay starting the treatment waiting for the culture results. Consequently, for the patient, anti-TB drugs (isoniazid, rifampin, ethambutol, and pyrazinamide) were started for 2 months, and then anti-TB drugs (isoniazid and rifampin) continued for 10 months. The 12-month duration was chosen due to the disseminated nature of bone marrow TB, the presence of caseating granulomas, and the patient's immunocompromised status from chronic corticosteroid use (5 mg prednisone daily), which increases the risk of relapse and necessitates extended treatment to ensure complete eradication of the infection [7]. Through this comprehensive approach, the medical team aimed to manage the patient's symptoms and improve his overall health status. The patient was visited every month, and pancytopenia was also corrected in CBC tests.

## 3. Discussion

Here, we presented the case of a 71-year-old man who presented to Loqman Hakim Hospital ED with significant weakness and fatigue, along with a chronic productive cough. His medical history included pituitary surgery and a regimen of 5 mg prednisone. Physical examination revealed a soft abdomen and no acute distress, but laboratory tests showed pancytopenia and hyponatremia. Despite normal peripheral blood smear results and vitamin B12 treatment, his cytopenia worsened. Further evaluations, including liver elastography and magnetic resonance imaging (MRI), were performed, and microbiological cultures returned negative. In this case, resource constraints and delays in biopsy processing prompted earlier imaging to assess organ involvement, guided by ultrasound findings of hepatic microabscesses and splenomegaly. A bone marrow biopsy revealed a granulomatous response with caseation, confirming MTB through acid-fast staining. He was subsequently started on anti-TB treatment.

Bone marrow TB is a rare but serious form of extrapulmonary tuberculosis that affects the bone marrow, the spongy tissue responsible for producing blood cells. It typically arises from hematogenous spread of MTB, which can occur in individuals with pulmonary TB or as a primary infection. Patients often present with nonspecific symptoms such as fever, night sweats, unexplained weight loss, and anemia [4]. Granulomas are reported in 0.3%–3% of bone marrow biopsies overall. In a high TB prevalence setting in China, a study of 11,339 bone marrow biopsies found granulomatous lesions in 0.97% of cases. Among these, TB was the most common etiology, accounting for 87.32% of infectious cases with confirmed diagnoses [4]. Clinical suspicion for disseminated TB involving the bone marrow should be raised by specific findings, including persistent fever, unexplained weight loss, night sweats, and pancytopenia (e.g., low white blood cell count, hemoglobin, and platelets), often with normocytic, normochromic anemia on peripheral smear [8]. Imaging findings such as pulmonary nodules, pleural effusions, splenomegaly, free abdominal fluid, and hepatic lesions suggestive of microabscesses (e.g., T1 hypointense/T2 hyperintense with peripheral enhancement on MRI) further support the diagnosis, particularly in immunocompromised patients or those with risk factors such as unpasteurized dairy consumption [9]. Bone marrow biopsy revealing caseating granulomas, confirmed by acid-fast staining or culture, is diagnostic, with treatment involving prolonged antitubercular therapy (6–12 months) [8]. A definitive diagnosis is often established through bone marrow aspiration or biopsy, where caseating granulomas may be identified. Treatment typically involves a prolonged course of antitubercular therapy, which can last up to 6 to 12 months [5].

TB caseating granulomas, marked by central necrosis, epithelioid macrophages, Langhans giant cells, and acid-fast bacilli on Ziehl–Neelsen stain, differ from other causes such as histoplasmosis (GMS-stained organisms, no Langhans cells), Nocardia (silver-stained filamentous bacteria), and cat-scratch disease (stellate necrosis) [4, 10]. Sarcoidosis typically shows noncaseating granulomas [10]. Clinically, TB presents with fever, night sweats, and weight loss, unlike sarcoidosis (e.g., uveitis) or fungal infections (travel-related) [4]. Absent granulomas on biopsy do not exclude TB, as atypical necrosis or focal lesions may occur [11]. Acid-fast stain has 72.7% sensitivity and 95.0% specificity in bone marrow biopsies [4].

Our knowledge of bone marrow TB is not that vast and in fact is limited to the few case reports written throughout the years. Tahir et al. reported a really similar case to the one report here. A 74-year-old Caucasian male with diabetes, chronic kidney disease, and COPD presented with weakness, low-grade fevers, and decreased appetite. Tests revealed pancytopenia and multiple centrilobular lung nodules. Initially suspected of TB, bone marrow biopsy confirmed miliary TB with bone involvement. He began a 6-month anti-TB treatment, leading to recovery [5]. In another case report by Dalugama and Gawarammana, the case of a 56-year-old Sri Lankan man, who presented with pyrexia of known origin with significant loss of weight and loss of appetite, is reported. Examination revealed anemia, leukopenia, and mild hepatosplenomegaly, while cultures and imaging were unremarkable. Initially treated with antibiotics, he was diagnosed with disseminated TB after bone marrow biopsy confirmed the presence of caseating granulomas. Following standard anti-TB treatment, his condition improved significantly, and he regained his premorbid health after 9 months of therapy [12]. Both of these cases mirror our case's immunocompromise, pancytopenia, and biopsy-confirmed TB, as an important risk factor.

Disseminated TB is significantly influenced by conditions that compromise the immune system. Key predisposing factors include HIV infection, which markedly increases the risk of TB progression and dissemination, especially in individuals with low CD4+ counts [13]. Chronic corticosteroid use also weakens immune responses, facilitating TB reactivation and spread [14]. Other immunosuppressive states, such as malnutrition, diabetes, and certain malignancies, further elevate susceptibility to disseminated TB [13].

While TB is the most prevalent cause of granuloma formation in the bone marrow [15], it is crucial not to overlook other potential differential diagnoses. Other common causes of bone marrow granulomas are hemophagocytic lymphohistiocytosis (HLH), sarcoidosis, lymphoma, *Cytomegalovirus* (CMV) or *Epstein–Barr virus* (EBV) infection, malignant neoplasms, and drugs [16]. HLH is a rare but potentially life-threatening complication that can occur in the context of disseminated MTB infections. The diagnostic criteria for HLH encompass severe cytopenias, hyperferritinemia, and the identification of hemophagocytosis in the bone [17].

Since there is not sufficient literature on the involvement of bone marrow by TB, it is advisable that review research be executed on the available data to compile a comprehensive understanding of the disease's manifestations, improve diagnostic accuracy, and inform treatment strategies for patients with TB-related bone marrow involvement.

## 4. Conclusion

In conclusion, this case underscores the challenges of diagnosing bone marrow TB, a rare but serious form of extrapulmonary TB. The 71-year-old patient presented with nonspecific symptoms, including weakness, fatigue, and a chronic productive cough. Despite normal laboratory results and empirical treatment, his condition necessitated further investigation, which ultimately revealed caseating granulomas and confirmed *MTB*. This case highlights the importance of considering TB in patients with unexplained hematological abnormalities, especially in those with compromised immune systems. The limited literature on bone marrow TB emphasizes the need for additional research to improve diagnosis and treatment strategies. By raising awareness and enhancing our understanding of this condition, healthcare providers can ensure timely intervention and better outcomes. The successful management of this patient with anti-TB therapy demonstrates the potential for recovery, reinforcing the need for heightened vigilance in recognizing and addressing TB-related diseases.

## Figures and Tables

**Table 1:** Lab data.

White blood cells (WBC) = 3 thousand cells/μL (4.5–11)	Hemoglobin = 7.3 mg/dL (13.5–17.5)	Platelets = 85 thousand cells/μL (150–450)
C-reactive protein (CRP) = 68.8 mg/L	Blood culture after 3 days: no growth	Peripheral blood smear (PBS): normocytic, normochromic anemia, leukopenia without dysplastic features, and thrombocytopenia without abnormal platelet morphology

## Data Availability

The datasets used and analyzed during this study are available from the corresponding author upon reasonable request.
